# Comparison of group counseling with individual counseling in the comprehension of informed consent: a randomized controlled trial

**DOI:** 10.1186/1472-6939-11-8

**Published:** 2010-05-14

**Authors:** Rajiv Sarkar, Thuppal V Sowmyanarayanan, Prasanna Samuel, Azara S Singh, Anuradha Bose, Jayaprakash Muliyil, Gagandeep Kang

**Affiliations:** 1 Department of Gastrointestinal Sciences, Christian Medical College, Vellore - 632 004, Tamil Nadu, India; 2 Department of Biostatistics, Christian Medical College, Vellore - 632 004, Tamil Nadu, India; 3 Community Health Department, Christian Medical College, Vellore - 632 004, Tamil Nadu, India

## Abstract

**Background:**

Studies on different methods to supplement the traditional informed consent process have generated conflicting results. This study was designed to evaluate whether participants who received group counseling prior to administration of informed consent understood the key components of the study and the consent better than those who received individual counseling, based on the hypothesis that group counseling would foster discussion among potential participants and enhance their understanding of the informed consent.

**Methods:**

Parents of children participating in a trial of nutritional supplementation were randomized to receive either group counseling or individual counseling prior to administration of the informed consent. To assess the participant's comprehension, a structured questionnaire was administered approximately 48-72 hours afterwards by interviewers who were blinded to the allocation group of the respondents.

**Results:**

A total of 128 parents were recruited and follow up was established with 118 (90.2%) for the study. All respondents were aware of their child's participation in a research study and the details of sample collection. However, their understanding of study purpose, randomization and withdrawal was poor. There was no difference in comprehension of key elements of the informed consent between the intervention and control arm.

**Conclusions:**

The results suggest that the group counseling might not influence the overall comprehension of the informed consent process. Further research is required to devise better ways of improving participants' understanding of randomization in clinical trials.

**Trial Registration:**

Clinical Trial Registry - India (CTRI): CTRI/2009/091/000612

## Background

The doctrine of informed consent is a cornerstone of ethical medicine, both in clinical and in research settings. However, research has shown that often participants do not understand all of the information required to make an educated choice [1-3]. Studies have shown that the participants' ability to recall facts differ with different methods of providing information [4-6], although retention of information is usually poor in most settings. There are profound difficulties concerning the understanding of risks, which is crucial information that patients need to comprehend to make appropriate decisions and act in what they believe to be their best interests [7]. The comprehension of informed consent is also often influenced by the socioeconomic background and the environment of the study participants [8].

Studies, particularly from developing countries, are often carried out in settings with individuals from different cultural backgrounds and education levels, thereby posing challenges in administering the informed consent [9]. The moral importance of appropriate and complete communication of information cannot be overemphasised in this context.

There are published quantitative studies on comprehension of informed consent by research participants in developing countries [10-15]. However, to the best of our knowledge, there are no studies on the efficacy of group counseling for administration of informed consent. Group counseling, followed by individual discussion of informed consent, might have advantages over individual counseling alone in allowing individuals in close-knit communities to use their support and decision making systems prior to enrolling in a trial. In a community-based survey involving low-income women, the participants preferred a 'group consent' process [16].

This study was conducted to assess whether participants subjected to group counseling recall the informed consent better than those subjected to individual counseling. Group counseling can be helpful in large-scale community-based studies, like vaccine trials, in terms of logistics and ease of administration of the informed consent process and of communicating with communities about the study during its conduct and after its completion.

## Methods

### Study area and population

This study was undertaken as part of a clinical trial on the effectiveness of nutritional supplementation on malnutrition in under-5 children in the Kaniyambadi block of Vellore district. The Community Health and Development (CHAD) of Christian Medical College, Vellore provides primary and secondary health care to all residents in the study area. In the year 2005, this area had 102,629 permanent residents, with a male/female ratio of 1:1.02. The adult literacy rate was 83.2% for males and 59.2% for females; 41.2% of the residents belonged to the low socio-economic status (SES), 38.0% to the middle and 20.8% belonged to the high SES category (CHAD, unpublished data).

A survey was conducted in 16 rural pre-schools (balwadis), run by CHAD in the study area, to identify children with malnutrition. Children attending these balwadis come from families with similar educational background and SES status (CHAD, unpublished data). The parents of these children were then approached to allow their child to participate in a study wherein they were individually randomized to receive either a nutritional supplementation and health education or health education alone for a period of three months. Blood samples were collected at baseline and towards the end of the study for the estimation of serum albumin, plasma zinc, plasma vitamin B-12, hemoglobin and red cell indices. Monthly anthropometric measurements were also obtained. Out of a total of 141 malnourished children identified from the balwadis, 128 (90.8%) children were enrolled following written informed consent given by their parents [17].

### Collection of data

To assess the efficacy of group informed consent, parents of the malnourished children were randomized to receive either group counseling or individual counseling prior to individual administration of the informed consent. The unit of randomization was a balwadi, i.e. parents of all children from a particular balwadi were assigned to a particular arm. Randomization was carried out by an independent statistician, who was not involved with administration of the informed consent or collection of data. The allocation sequence was provided in an opaque, sealed envelope and was opened on the day of recruitment for the specific balwadi. Prior to opening the envelope, the name of the balwadi was printed on its cover.

The informed consent was administered by two study nurses, well versed with the study protocol. Each nurse was assigned equal number of balwadis in the group and individual informed consent category. A check list was provided to ensure that the person administering the informed consent cover all relevant points in the document. The group informed consent was administered in the form of focus group discussions. Each group comprised a minimum of 4 and a maximum of 9 participants. Following the group counseling, participants were given approximately 10-15 minutes for discussion. The nurse acted as a facilitator in the discussions and clarified any questions or doubts raised by the group. Following this, soon after the discussion written informed consent was taken from the participating family representative. For the balwadis not receiving group counseling, parents were approached individually, and after discussion, written informed consent was taken from the participating family representative.

Approximately 48-72 hours following administration of the informed consent, each participating family representative was approached by a field worker, not involved with the informed consent process, and interviewed with the help of a structured questionnaire. The questions were primarily focused on assessing the respondent's recall of key elements of the informed consent, which were understanding the fact that his/her child was participating in a research study, recognizing the nature and purpose of the study, the risks and benefits of participation, random allocation to either intervention or control arm, the voluntary nature of participation and the freedom to withdraw at any point. Socio-demographic data were also collected at baseline. The interviewers (field workers) were blinded to the allocation group of the respondents. Verbal consent was obtained from all participants prior to administration of the questionnaire. Both the study on informed consent and the study on nutritional supplementation were independently evaluated and approved by the CMC Institutional Review Board.

Sample size was calculated considering knowledge of the study intervention as the primary outcome variable. Accounting for a 10% loss to follow-up the sample size was calculated to be 120. With an alpha error of 5%, this would have a power of 80% to detect a difference of 25% in the primary outcome variable between the intervention (group counseling) and the control (individual counseling) arms. We did not adjust for clustering as we expected a very low design effect, given the homogeneity in the socio-demographic and educational profile of our potential participants.

### Statistical Analysis

Data were entered in Epi-Info 2002 (CDC, Atlanta, GA, USA), and analyzed using STATA version 9.0 (StataCorp, College Station, TX, USA) software. Descriptive statistics were calculated for all study variables. The intracluster correlation coefficient (ICC) calculated for the present study was found to be 0.02. Consequently, the design effect was calculated as 1.1 (1.0-1.2), with a median (range) cluster size of 7 (3-12). This was considered to be low [18], hence, standard methods of analysis were used. Comparison between the intervention and control arm was done using the χ^2 ^test or the Fisher's exact test for categorical variables and using the two-tailed independent *t*-test or Mann-Whitney U test for continuous variables.

## Results

Of a total of 128 participants enrolled in the study, we could contact a total of 118 (92.2%) participating family representatives. The mean (SD) age of respondents was 29.3 (7.3) years. There was no significant difference between the intervention and control arm in terms of age of the respondents (*t*-test, *P *= 0.36). A large proportion of the respondents (104, 88.1%) were Hindus. Most were married females (113, 95.8%). Twenty-one (17.8%) of the respondents did not have any formal education and 101 (85.6%) did not finish high school (year 10). Almost half of the families (51, 44%) belonged to the low socio-economic status. The socio-demographic characteristics of the respondents are represented in Table 1. Overall, the intervention and control groups were comparable in terms of baseline socio-demographic characteristics.

**Table 1:** Baseline socio-demographic characteristics of the respondent families

Variable	Intervention (n = 50)	Control (n = 68)	*P*-value
Mean (SD) age of respondent ^1^	27.58 (6.76)	28.82 (7.61)	0.36
Mean (SD) age of study child ^1^	3.49 (0.96)	3.64 (0.84)	0.37
Respondent gender: Female ^2^	50 (100%)	65 (95.6%)	0.26
Child gender: Female^3^	27 (54%)	31 (45.6%)	0.37
Hindu religion ^2^	46 (92%)	58 (85.3%)	0.39
Mean (SD) years of education (respondent) ^4^	6.22 (3.05)	5.35 (3.63)	0.22
Mean (SD) years of education (head of the household) ^1^	6.56 (3.98)	6.74 (3.32)	0.80
Nuclear family ^3^	27 (54%)	34 (50%)	0.67
Housewives(only for female respondents) ^2^	37 (74%)	39 (60%)	0.12
Low SES ^2^	24 (48%)	27 (39.7%)	0.37
Mean (SD) number of family members ^1^	5.12 (1.53)	5.62 (2.18)	0.17

1. Comparison using t-test

2. Comparison using Fisher's exact test

3. Comparison using χ^2 ^test

4. Comparison using Mann-Whitney U test

All respondents knew that their child was participating in a research study. However, when asked what the main purpose of the study was, 113 (95.8%) of the respondents stated that it was to test how many children were underweight. Only one respondent could state the real purpose of the study, i.e. to test the efficacy of nutritional supplementation on underweight children. When asked about the study intervention, 111 (94.1%) respondents correctly identified either one of the two interventions i.e. special food supplementation or health education. There was no difference between the intervention and control arm in this respect (Fisher's exact test; *P *= 0.13). Only three respondents correctly identified both.

Everyone was aware of the fact that blood samples and monthly anthropometric measurements would be obtained from their child as a part of the study protocol. Most of the respondents (112, 94.9%), however, failed to comprehend the random nature of allocation of intervention with 99 (88.4%) stating that it was the balwadi teacher who would decide what intervention their child would receive. The proportion of such respondents were comparable across the intervention and control groups (Fisher's exact test, *P *= 0.15).

More than half of the respondents (73, 61.9%) did not perceive any risk to their child by participation in this study, although a larger proportion of respondents in the intervention group perceived some risk to their child [24 (48%) vs. 21 (30.9%)]; this difference was near significant (χ^2 ^test, *P *= 0.06). Almost all respondents (116, 98.3%) said that their child would benefit from this study, and all stated that the study would benefit other children in future. The most important anticipated benefit to their child was availability of free treatment or at a subsidized rate at the CHAD hospital (n = 107, 90.7%). This remained constant across both intervention and control group (Fisher's exact test, *P *= 1.00).

Sixteen (32%) respondents in the intervention arm and 19 (27.9%) respondents in the control arm (χ^2 ^test, *P *= 0.63) consulted either spouse (25, 22.1%) or parents (5, 4.4%) or both (5, 4.4%) before enrolling their children into the study.

When asked whether they felt compelled to join the study, 115 (97.5%) answered in the negative. However, only 54 (45.8%) know that they were free to leave the study at any point. Many respondents (95, 80.5%) felt that not participating in the study could adversely affect their or their children's regular medical care. These did not differ significantly between the intervention and control groups (χ^2 ^test, *P *= 0.12 and *P *= 0.73 respectively). Table 2 summarizes the result of the comparison of the respondents' understanding of the key elements of the informed consent between the intervention and control arm.

**Table 2:** Comparison of responses between the intervention and control group

Participants' knowledge and belief about study components	Intervention (n = 50)	Control (n = 68)	*P*-value
Study involving underweight children	48 (96%)	65 (95.6%)	1.00
Knowledge of either of the two study interventions	45 (90%)	66 (97.1%)	0.13
Randomization	3 (6%)	3 (4.4%)	0.70
Collection of blood samples	50 (100%)	68 (100%)	-
Anthropometric measurements	50 (100%)	68 (100%)	-
Benefit their own child	50 (100%)	66 (97.1%)	0.51
Benefit other children	50 (100%)	68 (100%)	-
Free treatment as a perceived benefit	45 (90%)	62 (91.2%)	1.00
Consulted others before deciding about participation	16 (32%)	19 (27.9%)	0.63
Joined the study voluntarily	49 (98%)	66 (97.1%)	1.00
No difference in child's medical care if not joining the study	9 (18%)	14 (20.6%)	0.82
Could leave study at any time without adverse consequences ^1^	27 (45%)	27 (39.7%)	0.12

1. Comparison using χ^2 ^test; other comparisons using Fisher's exact test

## Discussion

A true and meaningful informed consent is one of the cornerstones of ethical research. However, administering the informed consent in a manner in which it is easily comprehensible to the research participants is a major challenge for researchers in developing countries. Studies in different settings have found that participants' understanding of informed consent is poor [19-22]. Increasing pressure on researchers to recruit participants within a limited time-frame due to budgetary and other financial constraints has led to instances of unethical research practice, including improper administration of informed consent [23].

In order to improve the participants' understanding of the information provided, researchers have tried different methods to supplement the traditional informed consent process [5,6,11,24,25]. However, these interventions have shown conflicting results. A systematic review of trials on interventions to enhance participants' understanding of informed consent failed to find any evidence of positive association and concluded that further research was needed [4].

We undertook a randomized controlled trial to assess whether group counseling prior to administration of informed consent resulted in better comprehension of the informed consent than individual counseling. We felt that this would not only ease the burden of recruitment on researchers, but discussion among the probable participants could also help enhance their comprehension and help make a better decision, thereby improving the overall quality of the informed consent process. In this study, however, there was no difference in comprehension of key elements of the informed consent between the intervention and control group.

This apparent lack of difference between the intervention and control group could be due to many factors including the fact that a large proportion of our study population comprised mainly of people from the low SES and with lower literacy levels. Previous studies have shown that illiteracy and SES adversely affect a participant's comprehension of the informed consent [26-28], although, in a multicentric trial of a lipid lowering agent, researchers noted that the comprehension of the study participants did not differ by education or SES provided the consent form is explained in a simple language [29]. Using a simplified version of the written consent document with pictorial representation and the use of consent educators or professional nurses with prior research experience have also been shown to improve the participants' comprehension [30-32]. Devoting more time for explanations, use of the local language and obtaining consent at home have also been suggested as potential means to improve the informed consent process [33].

A major methodological limitation of this study was that it was not conducted across different studies. It has been shown that parents of children with acute life-threatening conditions find it more difficult to comprehend information than parents of children with less acute conditions [34]. Also, researchers have found that inability to concentrate at the time of signing the consent form could also adversely affect comprehension of the study procedures and outcomes [35]. Under such circumstances, the group consent process might be more effective as the participants are more likely to share information amongst them. A second limitation of this study was that all recruitments in a particular balwadi (for the study on nutritional supplementation) were done on the same day. The effect of intervention may have been diluted to some extent as the control group could possibly have discussed the research study. Further, although the study nurses were provided with a checklist to cover all the relevant points at the time of administration of informed consent, the researchers did not exercise any control over the discussions during the counseling session, either group or individual. As a result, we cannot know precisely what was discussed in the counseling sessions. This fact potentially limits our ability to evaluate comprehension solely on the basis of group vs individual informed consent process. In addition, studies conducted across a more diverse population group and in a more controlled environment might provide better results.

This study highlights limited comprehension about issues related to randomization and voluntariness among trial participants. Although the respondents knew that they were in a research study, the understanding of randomization and treatment allocation was poor. Previous research has also shown that in pediatric clinical trials, parents are less likely to understand the concepts of randomisation and this is more likely in people from the low socio-economic status [36]. Further research is required to devise better ways of improving participants' understanding of randomization in clinical trials.

## Conclusions

There was no difference in comprehension of the key elements of informed consent between participants who received group counseling and participants who received individual counseling to allow their children to participate in a trial of nutritional supplementation for malnourished children.

## Competing interests

The authors declare that they have no competing interests.

## Authors' contributions

All authors were involved in designing the study protocol and interpretation of the data. RS & PS analyzed the data. RS and GK wrote the manuscript. All authors read and approved the final version of the manuscript. RS and GK are guarantors of the paper.

## Pre-publication history

The pre-publication history for this paper can be accessed here:

http://www.biomedcentral.com/1472-6939/11/8/prepub
